# Animal Viruses, Bacteria, and Cancer: A Brief Commentary

**DOI:** 10.3389/fpubh.2014.00014

**Published:** 2014-02-13

**Authors:** Jimmy T. Efird, Stephen W. Davies, Wesley T. O’Neal, Ethan J. Anderson

**Affiliations:** ^1^Department of Public Health, Brody School of Medicine, East Carolina University, Greenville, NC, USA; ^2^Department of General Surgery, University of Virginia School of Medicine, Charlottesville, VA, USA; ^3^Department of Internal Medicine, Wake Forest University School of Medicine, Winston-Salem, NC, USA; ^4^Department of Pharmacology and Toxicology, Brody School of Medicine, East Carolina University, Greenville, NC, USA

**Keywords:** animal viruses, bacteria, epidemiology, cancer, infection

## Abstract

Animal viruses and bacteria are ubiquitous in the environment. However, little is known about their mode of transmission and etiologic role in human cancers, especially among high-risk groups (e.g., farmers, veterinarians, poultry plant workers, pet owners, and infants). Many factors may affect the survival, transmissibility, and carcinogenicity of these agents, depending on the animal-host environment, hygiene practices, climate, travel, herd immunity, and cultural differences in food consumption and preparation. Seasonal variations in immune function also may increase host susceptibility at certain times of the year. The lack of objective measures, inconsistent study designs, and sources of epidemiologic bias (e.g., residual confounding, recall bias, and non-randomized patient selection) are some of the factors that complicate a clear understanding of this subject.

## Introduction

Humans and animals have coexisted since the beginning of time, sharing viruses, bacteria, and perhaps the etiology of cancers. Approximately 75% of viruses and 50% of bacteria known to cause disease in humans are zoonotic and can be transmitted between animals and people (1). While evolution has provided adaptive immunity against microbes and cancer, the ability to defend against infection is sometimes absent or compromised. Excluding ionizing radiation, sunlight, and tobacco, infection represents the main known cause of human cancers throughout the world. The list is long including cancers of the anogenital track (HPV), stomach (*H. pylori*), liver (HBV, HCV, liver flukes), bladder (schistosoma hematobium), prostate (XMRV), and other specific cancers such as adult T-cell leukemia (HTLV-1), Kaposi sarcoma (HHV-8), Merkel Cell Carcinoma (MCPyV), and Burkitt’s lymphoma (EBV) (2, 3). The prevalence and persistence of tumor viruses varies in different parts of the world. Nearly 30% of cancers in developing and tropical countries are attributable to infectious causes compared with 10% in developed countries (4). However, the connection between viruses, bacteria, and cancer, and the role of animals, remains unclear or paradoxical in nature.

## Viruses

An infectious etiology for cancer was first documented in animals during the early part of the nineteenth century with the diagnosis of pulmonary adenocarcinoma in sheep (later attributable to jaagsiekte sheep retrovirus) (5). Animals are the host species for many oncogenes. Among the most studied are rodent (Abl, Int1/Wnt1, Int2, Notch1, Pim1/2, Runx, Tpl2), fowl (Erb-b, Fos, Myc, Src), feline (Myc), and fish (cyc) (6). For example, reticuloendothesliosis virus readily induces cancer in chickens (avian leucosis/sarcoma). The virus has been found in eggs intended for human consumption and vaccines prepared in eggs (7). A wide variety of viruses, mirroring their human analogs, are ubiquitous among animals in nature and their habitat (e.g., fecal coliform contamination) (8–10). Common types include viruses in the polyoma, adeno, retro, and papilloma family.

Animal viruses potentially express oncoproteins in human cells even though stringent replicate restrictions exist in the latter (11). The “hit and run” hypothesis posits that certain viruses interfere with the hosts immune system to cause cancer, yet do not integrate into the victims DNA (leaving no detectable fingerprints) (12). Newborn hamsters infected with polyoma virus have been shown to develop cancers, even though the cells of this species do not support virus replication (13). Similarly, tumors induced in immunocompetent mammals with Rous sarcoma virus do not present neutralizing antibodies (14). In contrast, some animal viruses [e.g., feline leukemia virus (FeLV)] have been observed to replicate *in vitro* in human cells (15, 16). Sera collected from 69% of 107 persons among 46 households with at least 1 FeLV gs-a positive cat tested positive for antibodies against FeLV (15). Although it is unclear exactly how antibodies directed toward animal viruses could have oncogenic or mitogenic effects on host cells, these findings support the idea that long-lasting “biological memory” of animal virus exposure can exist within the host in the absence of direct effects on host DNA.

## Bacteria

**Figure 1:**
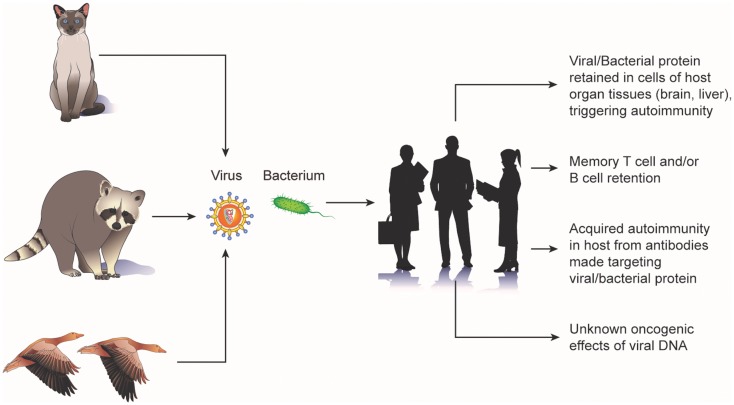
**Potential mechanisms of carcinogenic “biologic memory” retained from exposure to animal viruses and bacteria**.

Animal bacteria also have been implicated in cancer. The occurrence of gliomas in the brain of fowl have been noted in several reports (17–19) and these tumors have been described as having the pathognemonic encephalitic features of a pleomorphic parasite infection (e.g., hypertrophy and hyperplasia of blood-vessels; perivascular infiltration by lymphocytes, plasma cells, and monocytes; and the presence of A-D bodies) (20). Chickens spontaneously and experimentally infected with toxoplasma have been observed to develop glioma-like tumors (21, 22). A study of 16 human brain tumors observed bodies indistinguishable from the C and D phases of the fowl parasite (23). Epizootic outbreaks of toxoplasmosis have been reported in various avian species and mammals (22, 24, 25). Furthermore, toxoplasma antibodies have been isolated in the blood of exposed sheep farmers, flock animals, herder dogs, mice, and rats (26). Potential cellular mechanisms by which animal viruses and bacteria lead to tumorgenesis are shown in Figure 1.

## Age of Exposure

Early age at first exposure is critical for the development of many malignancies (27). Fetal or early childhood exposure to infectious agents occurs at a vulnerable time when the immune system is less developed and possibly more susceptible to tumor formation (28). The unrecognized persistence of an oncovirus or microbe in the host increases the chance for this agent to alter the cell cycle and immortalize cells (2, 29). Inoculation of immunologic immature neonate mice with polyoma virus results in tumor formation at multiple sites. In contrast, older mice do not develop tumors in response to polyoma virus either in the laboratory or by natural infection (13). The selective sensitivity of the fetus is further demonstrated in a study of patas monkeys wherein the administration of known carcinogenic agents in the fetal stage cause more tumors than an equivalent dose administered to juvenile monkeys (27, 30). Furthermore, few tumors developed when treatment was delayed until the start of the second trimester, suggesting that sensitivity was greatest during the first trimester.

## Transmission

### Exposure routes and factors influencing transmission

**Figure 2:**
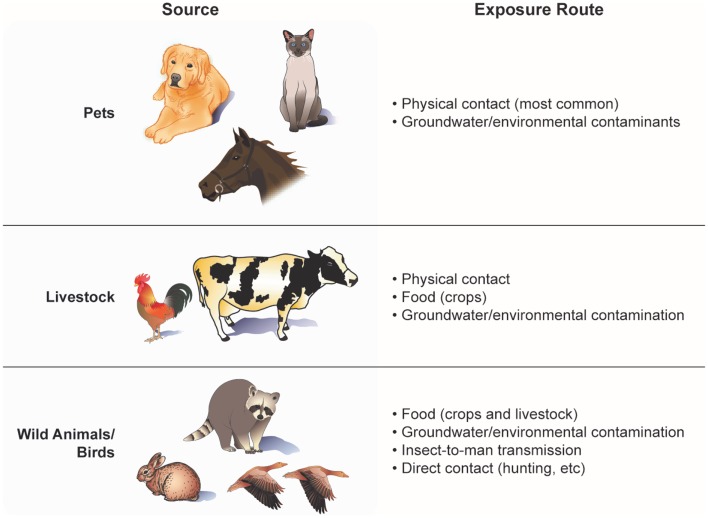
**Routes of transmission and human exposure to animal viruses and bacteria**.

Human exposure may occur in many ways – preparing and consuming animal products, washing with, and drinking well water contaminated with animal fecal coliform, animal bites/scratches, and working in occupations involving regular contact with animals, manure, soil, and/or by-products (e.g., farmers, slaughtering plant workers). Even living down-wind of a farm field fertilized with animal manure poses a potential risk. A list of major sources and exposure routes of animal-to-people transmission of viruses and bacteria is shown in Figure 2. Factors influencing the probability of disease transmission involve the proximity and temporal contact with the infectious organism, length of time that the infectious agent is present, virulence of the agent, incubation period, stability of the agent under varying environmental conditions, population density of carrier animals, husbandry practices, and control of wild rodents and insects (31). The type and maintenance of animal housing also may affect the extent to which individuals working in or around such facilities are exposure to zoonotic viruses and bacteria. Often, animal containment structures (e.g., hen houses, pig pens, cattle barns, and horse stables) may be inadequately ventilated and/or have poor waste removal systems, increasing the exposure of animals and their caretakers to dust, fecal matter, and microbes (32).

### Mechanical intermediate hosts

Infectious agents may be transmitted to humans by direct contact, fomite or mechanical vector, or intermediate hosts in which the agent multiples or develops before transmission to animal or human (i.e., metazoonoses). Examples of infectious agents requiring an incubation period prior to transmission include arboviruses, plague, and schistosomiasis (31). In the case of toxoplasmosis infection, contaminated soil and water represent a key source of infection emanating from an intermediate host (33, 34). Indoor/outdoor cats are a significant carrier/transmitter of Toxoplasma, shedding the organism in its feces (34). Oocysts from *Toxoplasma gondii* also may be transported by cockroaches and other bugs and deposited onto food and later consumed by animals and humans (35). In a recent study, eating raw oysters, clams, or mussels was identified as a new risk factor for *T. gondii* infection (36). The *T. gondii* were believed to have originated from cat feces, which survived or bypassed sewage treatment and traveled to coastal waters through river systems.

### Fetal infection routes

Many viruses and microbes are capable of crossing the placenta. For example, the TT circovirus and several polyomaviruses (BK, JC, and SV-40) are able to cross the placenta and infect the fetus, but do not cause critical fetal abnormalities (37). In the laboratory, influenza A virus has been detected in mouse pups born of mothers exposed to influenza A virus (38). Similarly, influenza RNA has been shown to persist for at least 3 months following birth of offspring to mice infected with influenza A/WSN/33 virus during pregnancy (39, 40). A fetus may become infected with varicella (chickenpox) from its mother in the early weeks of pregnancy, manifesting as congenital defects particularly involving the cerebellum (41–43). Human transplancental transmission is well demonstrated by cases of fetal infection with rubella virus (44), West Nile virus (45), toxoplasmosis (46), and bovine leukemia virus (47).

### Non-species-specific infection

Mice, rats, and other rodents potentially carry non-species-specific infectious diseases (31, 48, 49). Rodents also harbor insects and arachnids such as fleas, mites, and lice that can facilitate transmission of viruses, bacteria, and parasites to and between humans (31, 49). Certain arthropods in the mite family are known to transmit blood-borne diseases like “*Rickettsia typhi*” from mice and rats to humans (49). Although mites typically are host specific, they will cross from one species to another if their choice of host is unavailable (50, 51). Fleas are capable of passing diseases by blood from rodents to humans including bubonic plague, typhus, bartonellosis, and tapeworm (31, 48). Exposure to the respiratory or urinary aerosol of rodents also poses risk for human infections (e.g., *Hantavirus, Mycoplasma pulmonis, Streptococcus pneumoniae, Staphylococcus aureus*, and *Pasteurella multocida*) (52, 53).

The ease with which rodents transmit infection to humans likely reflects the high degree of homology between the two species. Approximately 80% of mouse genes have a single identifiable ortholog in the human genome, while <1% do not have any detectable human homolog (54). Occupations such as farming and agricultural work that place workers and their families in direct contact with rodents and their excretions/secretions increase the risk for infection (53, 55). KIPvV and WUPyV polyomaviruses have been detected in samples from children with lower respiratory tract disease, however it remains unknown if these viruses jumped species from rodents to humans (56).

### Indirect and other intermediate modes of transmission

Domestic farm animals and cats that feed on infected rodents or share habitat with reservoir species may indirectly expose humans to rodent-borne pathogens (31, 53). Antibody to the rodent Hantavirus has been found in cats (53, 57–60). A case-referent study conducted in China found a positive association between cat ownership and Hantavirus infection (61). In the case of orthopox infection, cats have been confirmed as an active transmitter from rodents to man (57, 59, 62–66). A prevalence of 4% was observed in an antibody study of orthopox infection in 200 free-roaming cats, most of which appeared to occur through bites sustained during hunting of rodents (59). Compared with other times of the year, a markedly increased incidence of feline poxvirus infection has been reported to occur during autumn, when the populations of many small mammals are at their largest and most active (67–69). Human infection from farm animals and cats/dogs typically results from direct contact with infected tissues, contaminated mucous membranes, and skin wounds; inhalation of respiratory secretions; and injuries sustained from infected animals (31).

Hantaviruses have been isolated from lung tissues of bats raising the possibility that these creatures also may pose a natural reservoir for the passing of rodent viruses to farm animals (53, 70). Bats appear to play a role as well in the infection of pigs with Menangle virus (71) and horses with Equine morbillivirus (72, 73). Notably, these mammals host a significantly greater number of zoonotic viruses per species than rodents (74). Bats also have been observed to have a greater number of interspecific “host switches” of paramyxoviruses to other mammals, compared with rodents, birds, primates, carnivores, and cetartiodactyla (74). Among humans, bats have been associated with the spread of severe acute respiratory syndrome-like corona viruses, Ebola and Marburg filoviruses, and Hendra and Nipah paramyxoviruses (74). Because of habitat encroachment and the proclivity of bats to roost in pitched roof spaces of buildings, humans are increasingly at risk for exposure to bat excreta (74).

Other intermediate modes for the transmission of infectious agents include insects and birds. Flying insects, for example, may feed on animal manure and later contaminate nearby food and water sources intended for human consumption, especially in the agricultural setting (48). Mosquitoes are known to transmit West Nile virus and Japanese B encephalitis from avian and horse hosts to humans (75–77) and conceivably may act in an analogous fashion as a carrier of oncogenic viruses between animals and humans. Lice, ticks, and other bloodsucking insects also can serve as mobile vectors to transfer neurotropic viruses (e.g., Alphavirus and Flavivirus) from reservoir host animals to other susceptible species including humans (31, 78). Wild ducks and migratory birds shed virus in their feces as they fly south in the fall, contaminating water on farms where chickens, turkeys, and pigs are raised (79).

### Low or negative antibody response

Viruses can induce cancer in species outside their natural hosts (80–82). However, not all viruses, especially those of avian origin, easily transmit directly to humans in nature (79). Canadian wildlife personnel, who handle wild ducks when they are shedding high levels of virus, consistently have negative viral and serologic assays (83). Volunteers inoculated with high doses of avian subtypes H4N8, H6N1, and H10N7 failed to produce a detectable antibody response, even though ~25% shed virus and had mild clinical symptoms (83). Only 2 of 27 persons in a sample of chicken farm laborers and residents regularly in contact with infected chickens and wildfowl tested positive for antibodies to Rous sarcoma virus-Bryan (RSV-B) (84). On the other hand, pigs have receptors for both avian and human viral strains and likely serve as a “mixing vessel” for genetic reassortment and subsequent introduction of genes into humans (85, 86).

### Direct human transmission

Avian viruses can directly infect humans without passing through pigs as evidenced by the recent outbreaks of influenza A strains H5N1 (87, 88) and H9N2 (89) in Asia, and H7N7 (90) in Netherlands (78, 91). The history of human infection closely parallels the domestication of the duck, which introduced respiratory-type influenza A viruses into the “farmyard (91).” H5 subtype infection initially was detected in human sera from rural China, a region known for poultry farming (87, 92). Approximately 30% of poultry workers sampled around the time of the 1999 H9N2 flu outbreak in Hong Kong were found to be seropositive for antibodies to the virus (89). H9N2 viruses are prevalent in domestic poultry such as chickens, ducks, geese, quail, and pigeons throughout Asia (89). In Netherlands outbreak, investigators reported a high infection rate of H7N7 among people directly involved in handling infected poultry and noted evidence for person-to-person transmission (90).

### Animal infection

Influenza viruses have been found to occur throughout the mammalian kingdom in nature (92, 93) and are capable of infecting (either naturally or experimentally) animals commonly found on farms including cattle, sheep, goats, horses, mice, and cats (53, 93–95). In Vietnam, civets (a cat-like species) have tested positive for H5N1 virus (96). Inoculation with various Influenza A strains (e.g., H5N1/97, H9N2, H5N3, A/tern/So, Africa/61, and A/turkey/England/63) can cause infection of mice (93, 97–100) and cross the blood–brain barrier (97–99, 101). The naturally occurring equine H7N7 influenza virus is highly pathogenic in laboratory mice, requiring no adaptation (99, 102).

### Species jumping

The transmission route for over 200 (~14%) human pathogens remains unknown and zoonotic transfer or species jumping cannot be ruled out as a potential source of infection (1). Among known routes, infected feces and urine remain one of the most prominent modes of transmission whether by primary or secondary sources. Animals that are not susceptible to infection may still spread disease to other animals and humans if they become contaminated and share water or food sources (103). However, for domestic poultry, secondary transmission often is associated with human involvement in the agriculture setting through personnel and/or animal movement, food delivery, and the use of fomites (103). Because poultry often do not display clinical signs of infection they may not be quarantined from other animals or their human caretakers (103). Furthermore, the increasing global demand for poultry and meat products coupled with improvements in transportation, have propagated closer contact between humans and farm animals (104).

## Epidemiology

### Lack of specific cancer risk across studies

Epiemiologic evidence in support of an animal-transmitted, viral or bacterial etiology for human cancers is limited and often inconsistent with respect to specific cancer risk. For example, exposure to farm animals or manure has been associated in some studies with childhood (105, 106) but not adult brain tumors (107, 108). A prospective cohort of 20,132 poultry, slaughterhouse workers (a group with frequent exposure to avian leucosis/sarcoma, reticuloendothesliosis, and Marek’s disease viruses) were observed to have a statistically significant excess of several cancers including lung [standardized mortality ratio (SMR) = 1.6, 95% CI = 1.3–1.7], cervix (SMR = 2.2, 95% CI = 1.3–3.5), penis (SMR = 8.6, 95% CI = 1.0–31.1), brain/nervous system (SMR = 1.7, 95% CI = 1.1–2.4), lymphoid leukemia (SMR = 2.2, 95% CI = 1.1–4.1), and monocytic leukemia (SMR = 9.2, 95% CI = 1.1–33.4) (7). Results of the Agricultural Health Study, a prospective cohort study of 49,884 male farmers, observed a statistically significant relative risk (RR) for non-Hodgkin lymphoma (RR = 1.6, 95% CI = 1.0–2.4) among farmers who raised poultry (109). However, in contrast to the above study of poultry, slaughterhouse workers, the RR among farmers who raised poultry was not significantly increased for either lung cancer or leukemia. Performing veterinary services also was associated with a statistically significant excess of Hodgkin lymphoma (RR = 12.2, 95% CI = 1.6–96.3) in the Agricultural Health Study. However, an increased risk for lymphoma among veterinarians has not been observed in other studies (110, 111).

### Viral exposure

In a medical record-based study of 83 cases and 166 referents individually matched on date of birth, sex, and hospital of birth, children of mothers who had documented evidence of a clinically diagnosed viral infection during pregnancy had an 11-fold odds ratio (OR) [confidence interval (CI) = 1.1–503.2] for childhood neoplasm of the brain compared with unexposed mothers (112). However, none of the noted viral infections (mumps, varicella, herpes zoster, and rubella) were related to animal exposure. Similarly, a 2.4-fold OR (CI = 1.5–4.0, 25 cases) for childhood brain tumors (CBT) was observed in a nested (within Swedish birth-cohorts 1973–1989) case-referent (545 cases, 2798 referents) study of children born to mothers who reported a wide variety of neonatal viral and bacterial infections during the pregnancy of the index child (113). Significantly increased risk estimates were specifically observed for CBT subtypes “low-grade astrocytoma” (OR = 2.7, CI = 1.2–5.8) and “high-grade astrocytoma” (OR = 5.0, CI = 1.0–24.8). Neonatal urinary tract infections were associated with a 7.5-fold OR (CI = 1.3–44.9) for low-grade astrocytoma. This is in contrast to other case-referent studies examining vaginal and genitourinary infections during pregnancy, which did not observe a statistically significant increased OR for CBT (106, 112, 114).

### Bacterial exposure

Cancer also has been linked with exposure to animal bacteria. In an ecologic, medical geographic study, a statistically significant 1.8-fold increase in brain cancer risk was observed for countries with increasing prevalence (4–67%) of *T. gondii* infection (115). In contrast, a case-referent study of brain cancer conducted in Australia observed no difference between participants with glioma (*n* = 117) and referents (*n* = 415) in the prevalence of antibody test-positivity (35% test-positive in glioma versus 33% in referents; age-, sex-, and center-adjusted OR = 1.00, 95% CI: 0.64–1.56) (33). While it is difficult to compare the results of the above two studies given their different study designs, ecologic studies generally are considered to provide the weakest form of epidemiologic evidence (116). For example, the ecologic link between brain cancer and *T. gondii* infection is difficult to interpret given the lack of patient-level information on histologic subtype and pathologic grade. Similarly, ecologic findings of increased cancer risk among dairy farmers must be carefully weighed against the absence of antibodies to bovine leukemia virus in these populations (117).

### Random cancer susceptibility

While few epidemiologic studies alone provide convincing evidence of an animal microbial basis for cancer, the data nevertheless are suggestive of a possible effect when examined as a whole. Perhaps the excess risk observed for some cancers, but not necessarily the same cancers across studies, may reflect a random cancer susceptibility to infection and inflammation rather than a specific microbe-cancer relationship. By analogy, animal viruses and bacteria may represent a chambermaid’s master key, capable of opening all hotel doors, but only if left unlocked by the guests.

## Explanation of Inconsistent Findings

Residual confounding or the lack of adjustment for factors such as population mixing, seasonality, climate, pesticides, medications, diet, and genetics may explain some of the conflicting and inconsistent results observed in the literature. Additionally, statistical results often are not appropriately adjusted for multiple comparisons and *post hoc* subset analyses, as was the case in a study reporting a statistically significant association between *T. gondii* infection and meningioma risk (33). Gene-environment interaction and the interplay between genes also are important puzzle pieces frequently missing from epidemiology studies of complex diseases such as cancer.

In the case of a “Hit and Run” virus, where the exposure theoretically occurred as an isolated event many years in the past, recall bias may have hindered the ability to establish causality or a temporal connection between a viral or bacterial exposure and cancer. A lack of study power, imprecision of point estimates, misclassification error, reverse causality, or selection bias represent other factors that may explain the inconsistencies observed across some studies and should be carefully considered with evaluating results (109, 112). Furthermore, the use of potentially carcinogenic disinfectant agents or cleaning compounds and must be taken into account as related etiologic exposures that might partially or fully explain a positive association between animal related microbial exposures and cancer.

## Conclusion

While the general population is commonly exposed to animal viruses and bacteria, many of which are known to cause cancer in animals, the etiologic role of these exposures in human cancer remains speculative. For example, animal oncoviruses generally are species specific and do not infect or replicate easily in humans. Nevertheless, animal viruses conceivably may cause cancer in humans analogous to human and simian polyomaviruses causing tumors in non-permissive rodents. Epidemiologic studies to date have provided little evidence that animal viruses and bacteria cause human cancer. Future studies will need to address the complex nature of cancer taking into account multiple interacting risk factors, and perhaps a non-stationary stochastic risk that contradicts conventional research design. The latter may be especially true given the waxing and waning behavior of viruses and bacteria. The same infectious agent may present and react differently depending on a host of factors including geography, seasonal variation and climate, population density, and herd immunity. Travel, hygiene, and cultural variation in food consumption and preparation among individuals further complicate the epidemiologic study in this field.

## Author Contributions

Jimmy T. Efird: conception of manuscript, drafting of manuscript, critical revision of manuscript, and final approval of manuscript. Stephen W. Davies: critical revision of manuscript and final approval of manuscript. Wesley T. O’Neal: critical revision of manuscript and final approval of manuscript. Ethan J. Anderson, critical revision of manuscript and final approval of manuscript.

## Conflict of Interest Statement

The authors declare that the research was conducted in the absence of any commercial or financial relationships that could be construed as a potential conflict of interest.
